# A face-to-face survey on the practice of ophthalmic clinicians in the management of dry eye disease in patients undergoing cataract surgery

**DOI:** 10.1177/11206721241312249

**Published:** 2025-01-21

**Authors:** Khayam Naderi, Laura Maubon, Chun Fung Jeffrey Lam, Harry Roberts, Vishal Shah, Darshak S. Patel, David O’Brart

**Affiliations:** 1 Department of Ophthalmology, St.Thomas' Hospital, Lambeth Palace Road, London, SE1 7EH, UK; 2 King's College London, Strand, London, WC2R 2LS, UK; 3 West of England Eye Unit, Royal Devon and Exeter NHS Foundation Trust, Barack Road, Exeter, EX2 5DW, UK; 4 University of Exeter Medical School, Heavitree Road, Exeter, EX1 2LU, UK

**Keywords:** Cataract surgery, dry eye disease, clinician survey

## Abstract

**Introduction:**

Dry eye disease (DED) can impact the accuracy of biometry measurements prior to cataract surgery (CS), influence visual performance post-CS, and can be exacerbated by CS. We performed a survey to evaluate the DED practice of clinicians directly caring for CS patients.

**Design:**

Prospective face-to-face survey.

**Method:**

Face-to-face survey consisting of 12 questions relating to CS clinicians’ estimations of DED pre- and post-CS, dry eye tests performed, and the management of DED.

**Result:**

There were one hundred and twenty-seven responders (39% consultants, 37% trainees/fellows, 8% associate specialists, 6% specialty doctors, 8% optometrists, 2% nurse specialists), with a 100% response rate. Sixty-seven percent routinely assessed for DED pre-CS, with 81% anticipating mild to moderative negative effects of CS on DED. Approximately 75% estimated that over 10% of pre-operative patients had asymptomatic DED, with another 10% or more suffering symptomatic DED. Almost 80% estimated that 10% or more of patients suffered DED post-CS. More DED tests were performed pre- compared to post-operatively (p = 0.02). More consultants performed dry eye tests post-operatively compared to non-consultants (p = 0.02). Most common treatment options included lubricating drops (95%), lid hygiene (75%) and night ointment/gels (54%). Seventy-six percent of surgeons performing CS stated they coated the ocular surface with an ophthalmic visco-surgical device and 34% limited intra-operative light exposure peri-operatively to limit DED.

**Discussion:**

Despite the anticipated negative effects of CS on DED, 1 in 3 clinicians in our survey were not assessing routinely for DED prior to CS, and fewer dry eye tests were performed post-operatively compared to pre-surgery.

## Introduction

Dry eye disease (DED) is a common multifactorial condition^1^ which can be exacerbated by cataract surgery (CS).^2–4^ Coupled with the high prevalence of patients with DED presenting for CS and the incongruence between signs and symptoms, the extent of evaluation and subsequent management of any pre-existing DED prior to surgery can be somewhat ambiguous for clinicians, especially when the patient is asymptomatic.^5–7^ The Tear Film and Ocular Surface (TFOS) Society Dry Eye Workshop (DEWS) II report recommends management of asymptomatic DED with positive signs prior to surgery where appropriate.^1^ This is particularly relevant in CS where treatment of pre-operative DED can optimise biometry measurements and reduce the risk of post-operative refractive errors.^8^

Because of the multifactorial and complex nature of DED, there are often variations in practice between clinicians in DED assessment and management.^9,10^ In addition, the lack of repeatability of DED clinical tests such as tear break-up time (TBUT), Schirmer I test (S1), and corneal and conjunctival ocular surface staining (OSS), can limit their potential interpretations and even their respective validities.^11^ At present there is limited evidence in the literature concerning the management of DED by clinicians prior to CS, and any peri-operative steps taken to limit its post-operative exacerbation.

To investigate current perceptions regarding DED and CS and its management, we conducted this face-to-face survey on the DED practice of clinicians in the United Kingdom (U.K) who directly care for CS patients.

## Methodology

**Figure 1.**
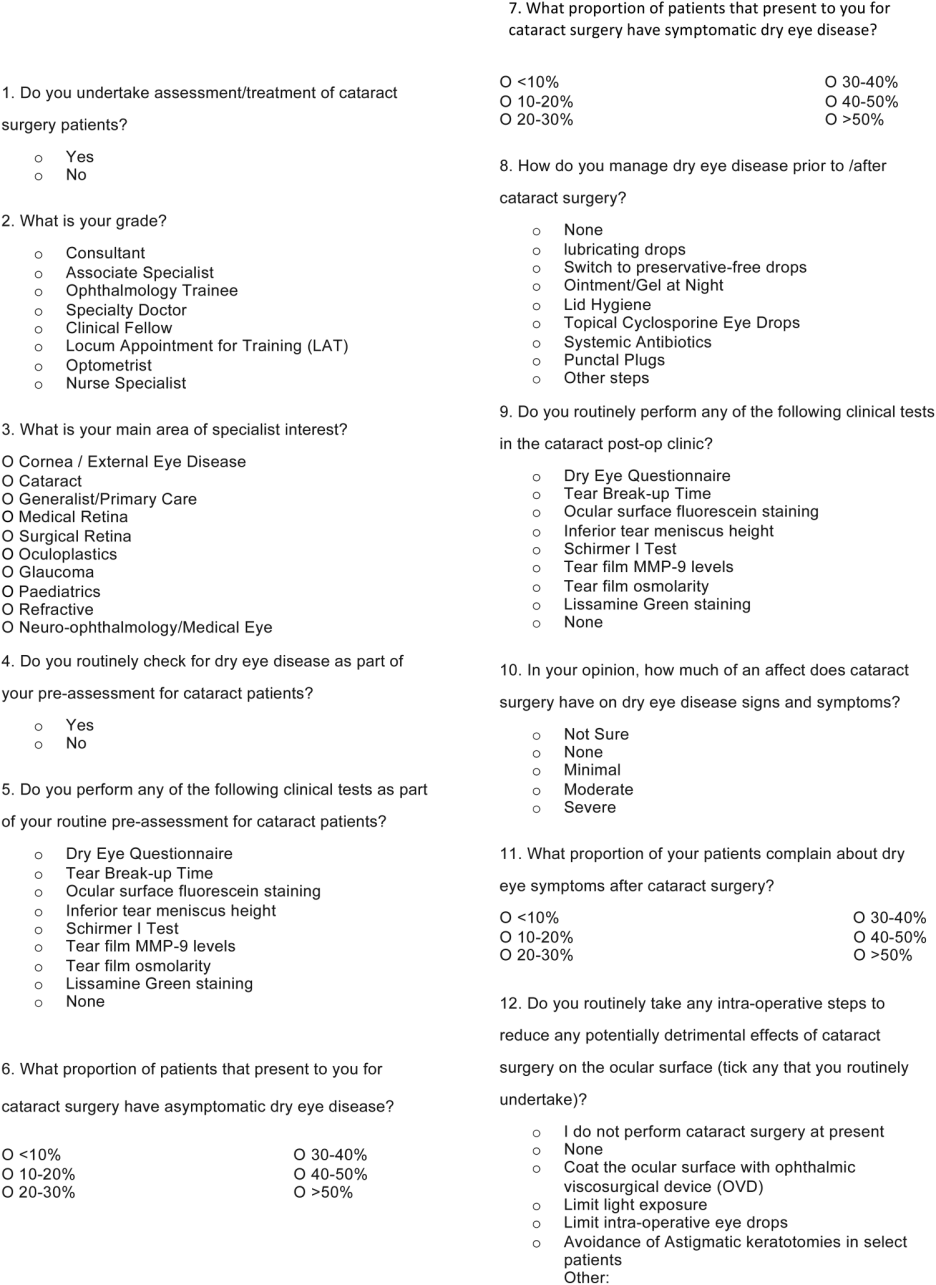
Dry eye disease in cataract surgery survey questions.

The survey was registered and approved by our local institution's audit and quality improvement project team. Data collection adhered to the tenets of the Declaration of Helsinki and the UK Data Protection Act (2018). Twelve questions (Figure 1) were constructed to evaluate the clinical assessment and management of DED in the pre- and post-operative CS clinics by clinicians in the U.K. Question 1 of the survey determined the eligibility of the clinician to participate in the survey, with questions 2 and 3 determining the clinician's training level and sub-specialty background in ophthalmology. Questions 4, 5, and 9 evaluated the extent of DED assessment performed on patients both prior to and after CS. Questions 6, 7, 10, and 11 considered estimates of the clinicians, based on their clinical experience, of the frequency of patients that are seen with signs and symptoms of DED pre- and post-CS, as well as the anticipated effects of CS on DED. Questions 8 and 12 assessed the management of DED and previously published peri-operative strategies to prevent DED exacerbation after CS.

The questions were asked as part of a face-to-face survey which was conducted opportunistically during two ophthalmology conferences in the UK, following permission from the conference organisers, and during local ophthalmology teaching sessions (following permission from the respective teaching leads) during the period 1^st^ February-31^st^ March 2020. Verbal consent was gained from each interviewee (clinician), with the respective answers anonymised. As the survey was prepared to be conducted opportunistically and in-person, the aim was to gain relevant information from as many clinicians as possible in a short space of time (approximate duration of 2–3 min per survey). The face-to-face element also allows interviewees to provide more instinctive responses to the provided questions. A small group of ophthalmology trainees conducted the face-to-face surveys, with the answers recorded on paper format. The anonymised raw data was then collected and stored into a Microsoft Excel (Microsoft, Redmond, Washington, USA) spreadsheet for analysis.

### Statistical analysis

Percentages of the survey responses were rounded up to the nearest whole number for consistency. Analysis of descriptive data was performed using Microsoft Excel (Microsoft, Redmond, Washington, USA). Statistical analysis was performed using GraphPad Prism version 8.0.0 for Mac (GraphPad Software, San Diego, California USA), with a Fisher's exact test used to compare proportions between groups. Statistical significance set at p < 0.05.

## Results

There were 127 survey responders (clinicians) who were eligible to participate in the survey as they responded yes to question 1 that they were currently undertaking assessment/treatment of CS patients. No clinician declined to participate in the survey, with a 100% response rate to the survey. Thirty-nine percent were consultant ophthalmologists, 37% were ophthalmology trainee doctors or fellows, 8% were associate specialists, 8% were optometrists, 6% were specialty doctors, and 2% were nurse specialists in ophthalmology. The responding clinicians worked in a range of sub-specialties, with 55 (43%) working in more than one subspecialty. The three most populated sub-specialties were cataract (57%), cornea and external eye disease (44%), and medical retina (26%). Sixty-seven percent of clinicians reported that they routinely checked for in patients prior to CS. With regards to the estimated negative post-operative effects of CS on DED signs and symptoms, 11% were unsure, 28% estimated the effects of CS to be mild, 53% estimated moderate consequences, and 8% estimated severe outcomes (Table 1).

**Table 1.** Clinician responses (n = 127) to their training grade, sub-specialty, whether they assess for dry eye disease in the cataract clinic prior to cataract surgery, and the potential negative effect of cataract surgery on dry eye disease.

Question	Clinician Response (n = 127)
Grade of Clinician	Consultant = 39% Associate Specialist = 8% Fellow = 13% Specialty Doctor = 6% Trainee = 24% Optometrist = 8% Nurse Specialist = 2%
Clinician Sub-specialty*	Cataract = 57% Cornea & External eye disease = 44% Medical Retina = 26% Generalist/Primary Care = 19% Glaucoma = 19% Oculoplastics = 9% Surgical Retina = 7% Neuro-ophthalmology/Medical Eye = 6% Refractive = 6% Paediatrics = 4%
Do you routinely check for dry eye disease as part of your pre-assessment for cataract patients?	Yes = 67% No = 33%
In your opinion, how much of an effect does cataract surgery have on dry eye disease signs and symptoms?	Not sure = 11% Mild = 28% Moderate = 53% Severe = 8%

*72/127 clinicians worked in one sub-specialty, 44/127 worked in two sub-specialties, 8/127 worked in three sub-specialties, 2/127 worked in four sub-specialties, and 1/127 worked in five sub-specialties.

Table 2 displays the estimated proportion of patients presenting to the cataract clinic with pre-operative asymptomatic DED, pre-operative symptomatic DED, and post-operative DED according to the responding clinicians. With regards to asymptomatic disease (where there is the presence of signs but absence of symptoms), 26% estimated that 10–20% of patients presented with asymptomatic DED, 3% were unsure and 9% of clinicians estimated that more than 50% of their patients had asymptomatic disease. Concerning symptomatic DED (where there is the presence of both signs and symptoms), 36% estimated that 10–20% of patients have symptomatic DED at presentation, 1% of clinicians were unsure and 6% felt that over 50% of their patients had symptomatic DED prior to CS. With regards to the estimated proportion of patients who present in the post-operative clinics with DED following CS, 18% felt that less than 10% present with DED, 26% stated that the incidence was between 10–20%, and 11% of clinicians thought that over 50% of their patients had DED post-CS.

**Table 2.** Clinician estimations (n = 127) of the proportion of patients presenting to the cataract clinic with asymptomatic pre-operative dry eye disease, symptomatic pre-operative dry eye disease, and post-operative dry eye disease.

Proportion of patients in the cataract clinic	Pre-operative Asymptomatic DED (n = 127)	Pre-operative Symptomatic DED (n = 127)	Post-operative DED (n = 127)
Unsure	3%	1%	2%
<10%	21%	24%	18%
10–20%	26%	36%	26%
20–30%	21%	17%	19%
30–40%	12%	9%	14%
40–50%	7.0%	6%	9%
>50%	9%	6%	11%

Percentages rounded up to nearest whole number.

DED = Dry eye disease.

With regards to specific clinical tests to evaluate for any DED, 27% of clinicians reported not performing any specific DED tests pre-operatively (Table 3). Of the clinicians who did perform dry eye tests, the top three most performed tests were ocular surface fluorescein staining (65%), TBUT (52%), and inferior TMH (25%). Post-operatively, 33% of clinicians reported not undertaking any specific DED testing. The three most performed tests post-CS were ocular surface fluorescein staining (59%), TBUT (39%), and inferior TMH (16%). There were no statistically significant differences between the number of each individual dry eye test performed pre- and post-operatively. There was a statistically significant difference in the total number of dry eye tests performed pre-operatively compared to post-operatively (192/889 vs 151/889, p = 0.02). There was no difference between consultants and non-consultants in the routine pre-operative assessment for DED (p = 0.44), or in the number of pre-operative dry eye tests (p = 0.22). Post-operatively, consultants performed a cumulative higher number of dry eye tests compared to non-consultants and this was statistically significant (p = 0.02) (supplementary table 1).

**Table 3.** Number of each dry eye test performed pre- and post-cataract surgery by the responders (n = 127).

Test Performed	Pre-operative	Post-operative	p-value
None	34/127 (27%)	43/127 (33%)	0.27
Fluorescein staining	83/127 (65%)	75/127 (59%)	0.37
TBUT	66/127 (52%)	50/127 (39%)	0.06
Inferior tear meniscus height	32/127 (25%)	20/127 (16%)	0.09
Dry Eye Questionnaire	5/127 (4%)	3/127 (2%)	0.72
Schirmer I Test	4/127 (3%)	1/127 (1%)	0.37
Tear film osmolarity	1/127 (1%)	1/127 (1%)	1.0
Lissamine Green staining	1/127 (1%)	1/127 (1%)	1.0
All tests	192/889 (22%)	151/889 (17%)	0.02

TBUT = Tear break-up time.

Fisher's exact test used to compare proportions of test(s) performed pre- and post-cataract surgery.

**Figure 2.**
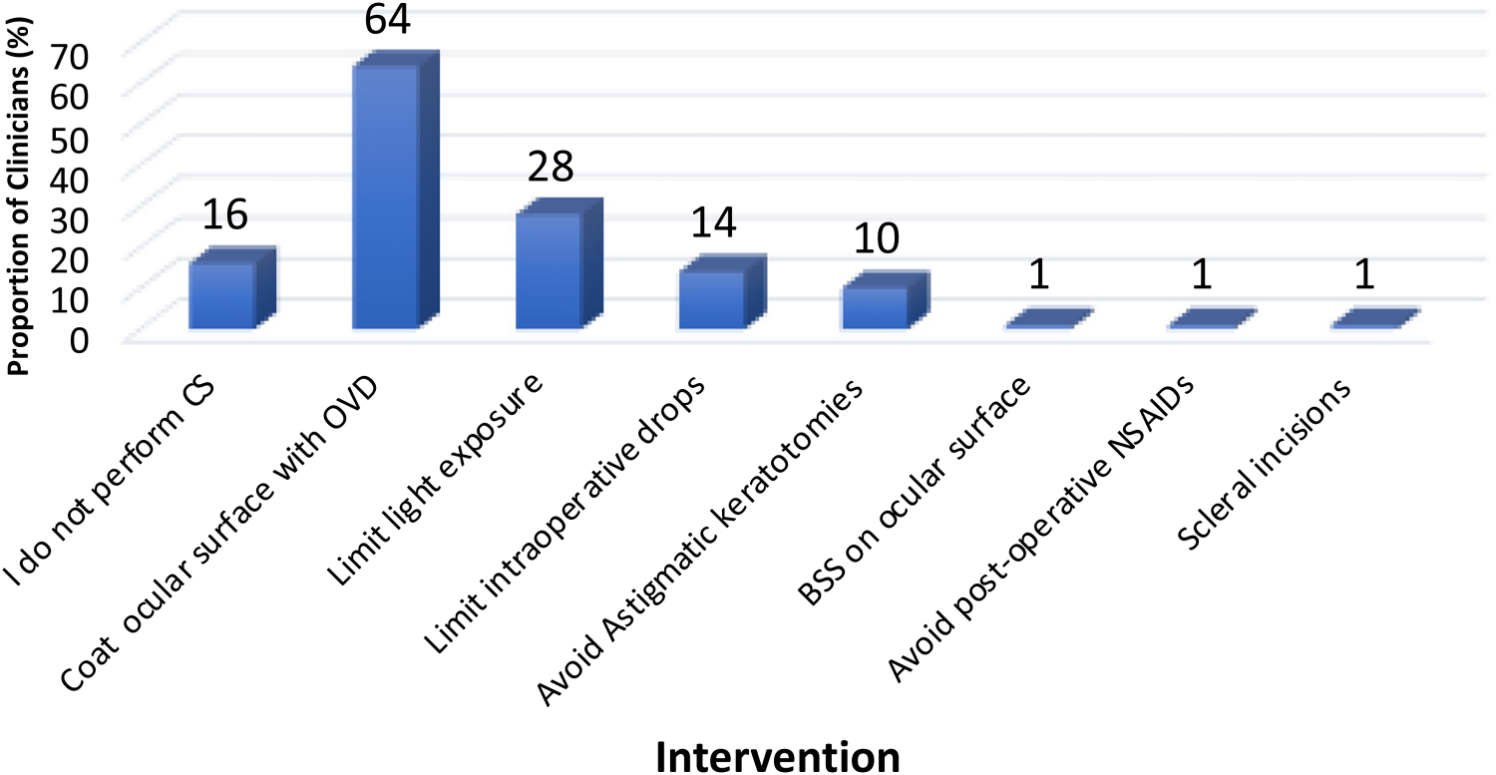
Bar chart showing the routine intra-operative steps performed by the responding clinicians (n = 127) to limit post-operative dry eye disease.

Figure 2 shows the routine steps taken peri- and post-operatively by clinicians to limit post-operatively DED signs and symptoms. Sixteen percent of clinicians did not perform CS. Of those that performed CS, the most reported practice shown to reduce DED post-CS was coating of the ocular surface during surgery with a dispersive OVD (76%). Further strategies included limiting intra-operative light exposure (34%), limiting intra-operative eye drops (17%), and the avoidance of astigmatic keratotomies in select cases (12%).

**Figure 3.**
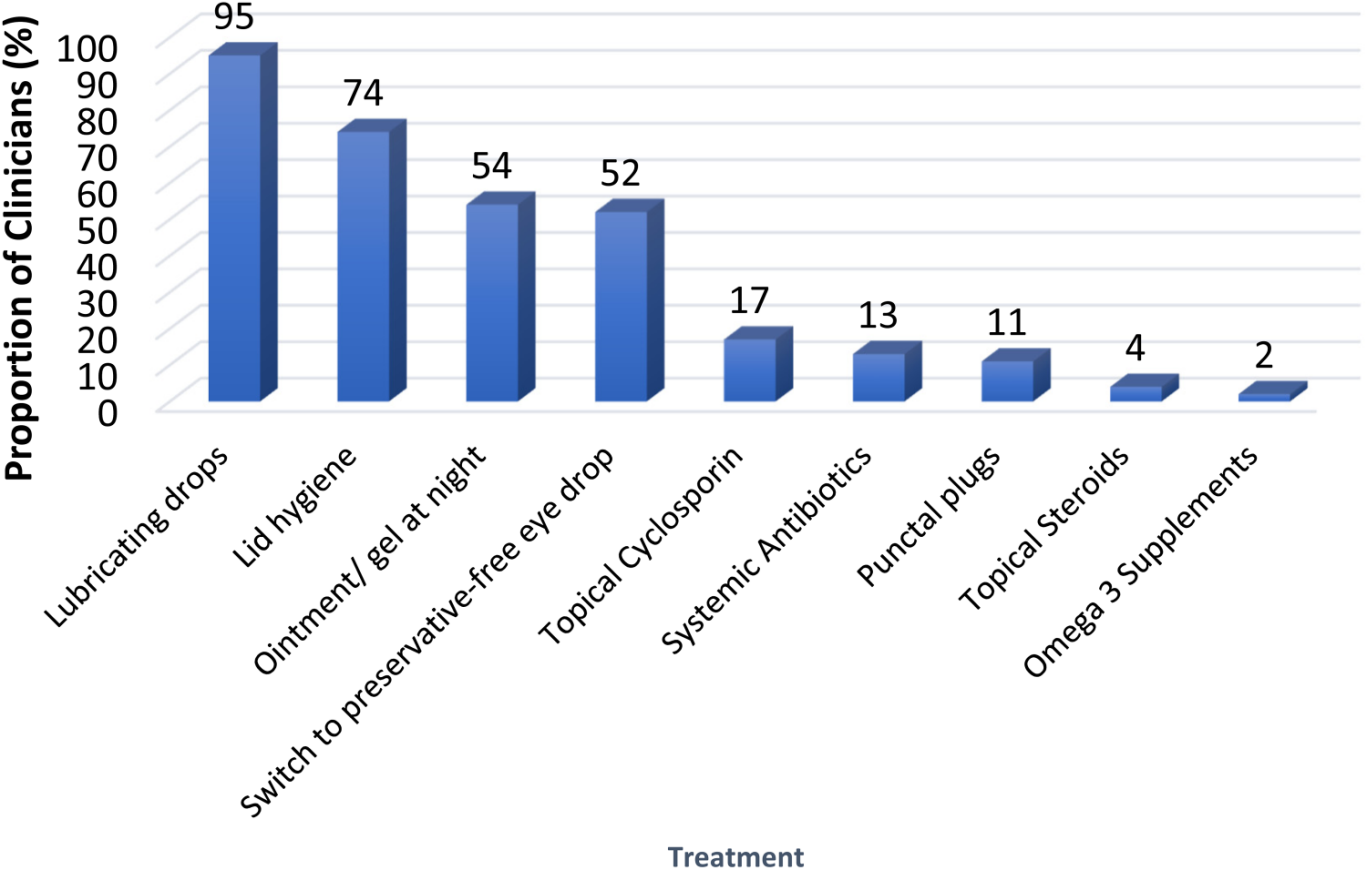
Bar chart showing the choice of treatment options prescribed by the responding clinicians (n = 127) for the management of dry eye disease (pre- and post-operatively).

Figure 3 shows the choice of treatments for the management of pre- and post-operative DED. Most clinicians (95%) prescribed lubricating eye drops, with 54% prescribing topical ointment/gel at night, and 52% switching to preservative-free eye drops post-CS. Seventy-four percent prescribed lid hygiene treatment and 17% topical cyclosporin. Other treatments included systemic antibiotics, punctal plugs, topical steroids, and omega-3 supplements.

## Discussion

In this survey, one-third of clinicians directly caring for CS patients, did not assess for DED in patients pre-CS, with 27% and 33% not performing any specific DED tests pre- and post-operatively, respectively (tables 1 and 3). These figures are low as the presence of DED is known to impact the quality of biometry calculations and can increase the risk of refractive surprise.^8^ The complex nature of DED may be a contributing factor to this variance in the assessment of DED, with often asymptomatic patients untroubled with DED symptoms, showing DED signs and anomalies on DED testing.^1,12^ As such, a biometry reading of apparent high reliability in asymptomatic patients referred for CS is unlikely to warrant the clinicians to carry out additional clinical tests to evaluate for potential DED. In addition, the time constraints posed by busy public health sector (National Health Service (NHS)) clinics with long waiting lists may also contribute clinicians to ‘focus’ on the patient's presenting complaint and not fully evaluate other co-morbidities. Indeed, in 2021–2022, there were 7.5 million outpatient attendances in ophthalmology clinics in the UK, with ophthalmology being the busiest specialty in the NHS.^13^

The discordance between the signs and symptoms of DED^6,7^ may also be a factor in how clinicians assess DED. Twenty-six percent of clinicians surveyed estimated that 10–20% of patients being assessed for CS had asymptomatic DED, with 36% estimating that 10–20% of patients have symptomatic DED. In contrast, in patients referred for CS, Gupta at al found at least one abnormal dry eye test in 80% of patients^5^ and Trattler et al^14^ reported 77% of patients to have an abnormal ocular surface staining prior to CS. Taking into consideration that one-third of our survey clinicians did not assess for DED prior to CS and 27% performed no specific DED testing, the prevalence of DED in patients presenting for CS is likely to be higher than the estimations of the survey cohort (table 3).

Despite studies showing the exacerbation of DED following CS,^2–4^ fewer dry eye tests were performed post-operatively compared to pre-operatively, with this difference being statistically significant (p = 0.02). This is surprising as 61% of our clinicians stated that CS has a moderate to severe negative impact on DED. Indeed, one study of patients undergoing phacoemulsification CS and manual small-incisional CS reported that all patients showed a deterioration in their DED parameters post-operatively,^4^ with another reporting that one-third of patients had persistent DED symptoms up to 6 months following CS.^2^ Interestingly, the consultant cohort in the survey performed more dry eye tests post-operatively (supplementary table 1) compared to non-consultants (p = 0.02). This suggests that post-operative effects of CS on DED are perhaps better appreciated by more experienced senior clinicians. The DED diagnostic tests recommended in the TFOS DEWS II report include symptomatology questionnaires, TBUT, tear osmolarity, and ocular surface staining.^15^ Interestingly pre-and post-operative utilisation of DED questionnaires was only 4% and 2%, respectively, with only a single clinician measuring tear osmolarity. This may suggest that there is limited access to tear osmolarity diagnostic equipment, and some clinicians may also be unaware the relevance of tear osmolarity in the context of DED. The lack of use of DED questionnaires may partly be due to time constraints in clinic. A limitation of our survey includes the omission of patient history and DED risk factors as part of the pre- and post-operative assessment. This may have been, in part, how evaluation of patient DED symptomatology was evaluated in place of the formal completion of DED questionnaires.

Although two-thirds of our clinicians assessed for DED prior to CS, most of those performing CS undertook peri-operative strategies to help limit DED problems post-CS. Over three quarters of the operating clinicians routinely coated the ocular surface with a visco-dispersive OVD (figure 2), although this step may also be undertaken to aid visualisation during surgery.^16^ Over one-third stated they limited intraoperative light exposure, although this step may also be undertaken by many surgeons to limit intra-operative retinal phototoxicity.^17^ It is one limitation of this current study that we did not specifically ask why these steps were undertaken by individual surgeons and were there reasons other than DED why they were performed. Interestingly, with regards to limiting post-CS DED by reducing any corneal nerve damage intra-operatively, 12% stated they limited astigmatic keratotomies in select patients and 1% performed scleral incisions rather than corneal incisions.

With regards to treatment of DED prior to and after CS, as might be expected with 96% and 54% of clinicians prescribed lubricating eye drops and night ointment and gel, respectively, with the treatment of underlying blepharitis being popular with 74% counselling lid hygiene treatment and 13% prescribing systemic antibiotics. These findings are comparable to the responses of clinicians in an online international survey on the general management of DED,^18^ although our survey is specific to DED management in the pre- and post-operative cataract clinics. Over 50% of respondents also suggested switching to preservative free drops for the management of DED problems (figure 3).

The advantages of a face-to-face survey (compared to an online survey) include a certain level of flexibility and fluidity in data collection, as well as allowing the establishment of rapport between the interviewer and interviewee.^19^ The non-response rate may be lower than online surveys, with a study by Cunningham et al^20^ showing a response rate of only 35% by physicians to an online survey. Furthermore, a study by Christensen et al^21^ also showed the non-response rate to a face-to-face survey being lower than self-administered questionnaires (23.7% vs 37.9%). However, the limitations of face-to-face surveys must also be acknowledged, including the influence of social desirability bias when interviewees provide their respective answers, as well as time pressures to complete the survey promptly.^22,23^

There are limitations to this study. When constructing the 12 questions, a balance had to be made between gaining appropriate information from clinicians, whilst avoiding the prolonging of each survey to limit non-response rates. Therefore, additional questions would have been helpful in further exploring clinician beliefs and practices in relation to DED and CS. An additional question which may have provided valuable information include ‘what is your threshold for commencing treatment for DED prior to CS?’. This question would particularly be useful in exploring the practice of the cohort of clinicians in the survey who do not routinely look for DED in patients pre-operatively. Another potential useful set of questions would be the clinicians’ views on the reliability and repeatability of the various dry eye tests. This may have shed further light on why each clinician chose to perform certain dry eye tests as opposed to others. A limitation of question 10 of the survey is the clinician's subjective interpretation of ‘mild’, ‘moderate’ and ‘severe’ with regards to the negative effects of CS on DED. However, grading DED can be challenging due to the plethora of dry eye assessments and symptomology questionnaires,^1^ and the inconsistent relationship between the clinical signs and symptoms of the condition.^24^ This study was also commenced just prior to the COVID-19 pandemic which due to the imposition of lockdown restrictions curtailed face to face meeting and the number of clinicians we were able to survey.

## Conclusions

In this face-to-face survey of practising ophthalmology clinicians in the UK with direct care of CS patients, there was a wide range in the assessment and management of DED prior to and following CS. Despite the literature suggesting a deterioration in DED parameters and symptomatology post-CS,^2,20^ one-third of clinicians did not assess for DED prior to CS, with fewer dry eye tests performed in the post-operative review of CS patients compared to pre-operatively. The findings from the clinician cohort in this survey suggests that there may be significant variability in practice amongst clinicians with regards to DED evaluation and management in relation to CS, and further highlights how the complexity of DED may have limited the development of an overarching clinical approach to its evaluation and subsequent management. Further research is required in this field, including studies to identify the sensitivity and specificities of the various dry eye tests, and randomised control trials on the benefits of various DED treatments pre- and post-CS.

## Supplemental Material

sj-docx-1-ejo-10.1177_11206721241312249 - Supplemental material for A face-to-face survey on the practice of ophthalmic clinicians in the management of dry eye disease in patients undergoing cataract surgery Supplemental material, sj-docx-1-ejo-10.1177_11206721241312249 for A face-to-face survey on the practice of ophthalmic clinicians in the management of dry eye disease in patients undergoing cataract surgery by Khayam Naderi, Laura Maubon, Chun Fung Jeffrey Lam, Harry Roberts, Vishal Shah, Darshak S. Patel and David O'Brart in European Journal of Ophthalmology
