# Supplementary material for: A face-to-face survey on the practice of ophthalmic clinicians in the management of dry eye disease in patients undergoing cataract surgery
Source: Eur J Ophthalmol. 2025 Jan 21;35(4):1195–202. doi: 10.1177/11206721241312249 (PMC12166159; doi:10.1177/11206721241312249)
Supplement: sj-docx-1-ejo-10.1177_11206721241312249 - Supplemental material for A face-to-face survey on the practice of ophthalmic clinicians in the management of dry eye disease in patients undergoing cataract surgery [file sj-docx-1-ejo-10.1177_11206721241312249.docx]

|  | Consultants  (n=49) | Non-consultants (n=78) | p-value |
| --- | --- | --- | --- |
| Routine assessment for pre-operative DED  (Yes: No) | 35:14 | 50:28 | 0.44 |
| Pre-operative DED tests | 93/343 | 99/546 | 0.22 |
| Post-operative DED tests | 71/343 | 80/546 | 0.02 |
| Supplementary Table 1: Comparison of consultants vs non-consultants with regards to routine assessment for pre-operative dry eye disease, completion of pre-operative dry eye tests, and completion of post-operative dry eye tests.  Seven dry eye tests available for the 49 consultants in the survey (7x49) and the non-consultants (7x78) pre-operatively and post-operatively.  Fisher’s exact test used to compare proportions.  DED=Dry eye disease. | | | |
